# Effectiveness of catheter ablation of atrial fibrillation according to heart failure etiology

**DOI:** 10.1002/joa3.12291

**Published:** 2020-01-08

**Authors:** Eric Black‐Maier, Benjamin A. Steinberg, Kevin M. Trulock, Frances Wang, Yuliya Lokhnygina, Wanda O'Neal, Sana Al‐Khatib, Brett D. Atwater, James P. Daubert, Camille Frazier‐Mills, Donald D. Hegland, Kevin P. Jackson, Larry R. Jackson, Jason I. Koontz, Robert K. Lewis, Albert Y. Sun, Kevin L. Thomas, Tristram D. Bahnson, Jonathan P. Piccini

**Affiliations:** ^1^ Cardiac Electrophysiology Section Duke Center for Atrial Fibrillation Duke University Medical Center Durham NC USA; ^2^ Department of Biostatistics Duke University Durham NC USA; ^3^ Electrophysiology Section University of Utah Salt Lake City UT USA

**Keywords:** atrial fibrillation, cardiomyopathy, catheter ablation, heart failure, outcomes

## Abstract

**Background:**

Catheter ablation is an important rhythm control therapy in patients with atrial fibrillation (AF) with concomitant heart failure (HF). The objective of this study was to assess the comparative efficacy of AF ablation patients with ischemic vs nonischemic heart failure.

**Methods:**

We conducted a retrospective, observational cohort study of patients with HF who underwent AF ablation. Outcomes were compared based on HF etiology and included in‐hospital events, symptoms (Mayo AF Symptom Inventory [MAFSI]), and functional status (New York Heart Association class) and freedom from atrial arrhythmias at 12 months.

**Results:**

Among 242 patients (n = 70 [29%] ischemic, n = 172 [71%] nonischemic), patients with nonischemic cardiomyopathy were younger (mean age 64 ± 11.5 vs 69 ± 9.1, *P* = .002), more often female (36% vs 17%, *P* = .004), and had higher mean left‐ventricular ejection fraction (47% vs 42%, *P* = .0007). There were no significant differences in periprocedural characteristics, including mean procedure time (243 ± 74.2 vs 259 ± 81.8 minutes, *P* = .1) and nonleft atrial ablation (17% vs 20%, *P* = .6). All‐cause adverse events were similar in each group (15% vs 17%, *P* = .7). NYHA and MAFSI scores improved significantly at follow‐up and did not differ according to HF etiology (*P* = .5; *P* = .10‐1.00 after Bonferroni correction). There were no significant differences in freedom from recurrent atrial arrhythmia at 12‐months between ischemic (74%) and nonischemic patients (78%): adjusted RR 0.63, 95% confidence interval 0.33‐1.19.

**Conclusions:**

Catheter ablation in patients with AF and concomitant heart failure leads to significant improvements in functional and symptom status without significant differences between patients with ischemic vs nonischemic HF etiology.

## Graphical abstract

Catheter ablation is an important rhythm control therapy in patients with atrial fibrillation (AF) with concomitant heart failure (HF). The objective of this study was to assess the comparative efficacy of AF ablation patients with ischemic versus nonischemic heart failure. We found that catheter ablation in patients with AF and concomitant heart failure leads to significant improvements in functional and symptom status without significant differences between patients with ischemic versus nonischemic HF etiology.

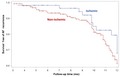

## 1 INTRODUCTION

Atrial Fibrillation (AF) and heart failure (HF) are two of the most common cardiovascular diseases. They both reduce quality of life and longevity, particularly when they accompany one another.^1^, ^2^ AF is independently associated with worse left ventricular systolic function and worse quality of life in patients with HF.^3^, ^4^, ^5^ Furthermore, the presence of HF complicates treatment for AF, as several antiarrhythmic drugs are contraindicated because of the potential for harm in HF. Yet, the remaining guideline‐advocated medical therapies, including dofetilide and amiodarone, suffer from suboptimal effectiveness and still have potential for harm. Drug toxicity is likely partly attributable for the failure of a “rhythm control” strategy to prove superior to rate‐only control in AF patients with or without HF.^6^, ^7^ Therefore, catheter ablation represents an appealing approach to the management of AF in these patients.^8^, ^9^, ^10^, ^11^, ^12^, ^13^.

Catheter ablation of AF is currently recommended for patients with symptomatic AF refractory to antiarrhythmic therapy.^14^ It has proven to be an effective therapy in patients with HF,^15^ resulting in improved freedom from AF, functional status and left ventricular function.^11^, ^12^, ^13^ In the recently published randomized controlled trial Catheter Ablation for Atrial Fibrillation with Heart Failure (CASTLE‐AF), AF ablation in patients with systolic HF led to statistically significant reductions in hospitalization and mortality.^16^ It is well appreciated that the etiology of cardiomyopathy and differences in underlying substrate influence outcomes in both catheter ablation of ventricular tachycardia and cardiac resynchronization therapy.^17^, ^18^ However, few studies have assessed the impact of HF etiology on outcomes of catheter ablation for concomitant AF. Accordingly, we sought to compare outcomes of AF ablation in patients with cardiomyopathy that was ischemic in origin, vs those with nonischemic cardiomyopathy. We hypothesized that patients with nonischemic cardiomyopathy may experience worse outcomes following AF ablation compared with patients with ischemic cardiomyopathy, because of a propensity for more extensive atrial myopathy.

## 2 METHODS

We conducted a retrospective observational cohort study within the Duke Center for Atrial Fibrillation. Consecutive AF catheter ablation procedures in adult patients (≥18 years) at the Duke University Medical Center from January 1, 2007 and June 30, 2013 were reviewed for inclusion in the analysis cohort. Only patients with a baseline clinical diagnosis of HF were included, and heart failure was defined clinically by the patient's primary cardiologist based upon signs and symptoms at the time of their HF diagnosis.^19^, ^20^ This included both HF with reduced and preserved ejection fraction. For the purpose of this analysis, the cohort was stratified by ischemic and nonischemic etiology. This was also defined by the patient's primary cardiologist, as to whether coronary artery disease, if present, was the predominant contributor to the patient's HF. Coronary artery disease was defined as an epicardial stenosis of 70% or greater (>50% in the left main coronary artery). Moreover, HF etiology was also confirmed with cardiovascular imaging (focal wall motion abnormalities on echocardiography and/or scar pattern on cardiac MRI). Hybrid catheter‐based and/or surgical procedures (open or thoracoscopic) and those using cryoballoon or laser ablation were excluded. Thus, for the purpose of this analysis, only radiofrequency ablation procedures were included. All procedures were performed under general anesthesia. Heparin was administered at the time of trans‐septal puncture and activated clotting times were maintained between 300 and 400 seconds. Ablation was performed with open‐irrigated catheters and the use of an electroanatomic mapping system (CARTO (Biosense‐Webster Inc, Diamond Bar, CA) or NavX (St. Jude Medical, Minneapolis, MN)). Intracardiac ultrasound was used in all cases. In all cases, pulmonary vein isolation was performed using a circumferential approach with documentation of entrance and exit block with the use of a circular decapolar catheter. Additional ablation lesions were performed at the discretion of the primary operator, and were most commonly driven by clinical circumstances. Anticoagulation was continued for a minimum of 3 months postprocedure and thereafter according to guideline recommendations based upon the CHA_2_DS_2‐_VASc score.

For each ablation, baseline demographics, medical history, imaging, laboratory data, and medical therapies were reviewed and abstracted. The diagnosis of obstructive sleep apnea was determined by polysomnography demonstrating apnea hypopnea index > 15 or apnea hypopnea index 5‐14 with suggestive symptoms.^21^ The index operative report was reviewed and abstracted as well. In‐hospital, periprocedural outcomes, as well as arrhythmia outcomes up to 12 months were recorded. Adverse events included any access site adverse events, pericardial tamponade, stroke or transient ischemic attack (TIA), acute HF, or in‐hospital death. Symptom status was assessed using the New York Heart Association HF classification scale, and using an abbreviated form of the disease‐specific Mayo AF Symptom Inventory.^22^ Clinical outcomes at last follow‐up included use of antiarrhythmic therapy, symptomatic recurrence, and documented arrhythmia recurrence. These assessments were based on clinical follow‐up of reported symptoms and arrhythmia recurrence was defined as: atrial tachycardia, atrial flutter, or atrial fibrillation (AT/AF/AFL) on a 12‐lead electrocardiogram; AT/AF/AFL ≥ 30 seconds on a continuous monitor or implantable device; or AT/AF/AFL that required cardioversion. We employed a 3‐month blanking period for the arrhythmia endpoint. In addition to chart review of primary data, assessment of symptoms and adverse events was performed by direct patient phone call starting 1‐week postprocedure, with an additional call at 3, 6 and 12 months, in addition to scheduled clinic visits. Symptoms were assessed using the modified Mayo AF‐Specific Symptom Inventory (MAFSI) questionnaire, which was administered in clinic preprocedurally and by phone call postprocedurally. Electrocardiograms were performed at regularly scheduled clinic visits at 3, 6, and 12 months. Additional ECG monitoring was performed at the discretion of individual providers and in the presence of symptoms concerning for arrhythmia recurrence. Patients with an implanted device received routine quarterly device interrogation including assessment for arrhythmia recurrence.^22^

### 2.1 Statistical methods

Baseline and ablation characteristics were described using counts and percentages (categorical) or mean and standard deviation (continuous). Univariate comparisons of baseline and ablation characteristics were made using Chi‐squared or Fisher's exact test for categorical variables and ANOVA for continuous variables as appropriate. Changes in NYHA classification between baseline and the latest follow‐up were analyzed using Wilcoxon signed rank test within each HF etiology group; changes were compared between the groups using Wilcoxon rank‐sum test.

Comparisons of in‐hospital and 12‐month outcomes between HF etiology groups were performed using Chi‐square tests. For 12‐month outcomes of freedom from recurrent atrial arrhythmia (AT/AF/AFL), adjusted logistic regression analyses were also conducted, where adjustment variables were selected among baseline characteristics using a parsimonious forward selection process with entry criteria of *P* < .25. Time to recurrent atrial arrhythmia was illustrated with the use of Kaplan‐Meier plots of event‐free survival.

This study was approved by the Duke University Institutional Review Board, which granted a common rule exemption to the requirement of individual patient informed consent. All statistical analyses of the aggregate, deidentified data were performed by the Duke Clinical Research Institute using SAS software (version 9.4, SAS Institute). *P* < .05 was considered statistically significant.

## 3 RESULTS

### 3.1 Baseline characteristics of the cohort

A total of 230 patients with a diagnosis of clinical HF underwent 242 catheter ablations for AF at Duke University Medical Center during the study period (n = 70 ischemic [29%], n = 172 nonischemic [71%]). There were important differences in baseline characteristics between the two groups, including age (mean 69 for ischemic vs 64 for nonischemic, *P* = .002), gender (female 17% for ischemic vs 36% for nonischemic, *P* = .004), and mean left‐ventricular ejection fraction (LVEF, 42% for ischemic vs 47% for nonischemic, *P* = .0007, Table 1). At baseline, there were no major differences in medical therapy between the two groups—patients not on antiarrhythmic therapy comprised 40% of the ischemic group and 35% of the nonischemic group (*P* = .28). Ablation procedure characteristics are shown in Table S1 and were roughly balanced between ischemic and nonischemic patients, including mean procedure times (259 minutes for ischemic vs 243 for nonischemic, *P* = .13), mean ablation time (58 minutes for ischemic, 56 for nonischemic, *P* = .6), and mean fluoroscopy time (51 minutes for ischemic vs 54 for nonischemic, *P* = .44). The most common adjunctive ablation, beyond PVI, was a left atrial roof line in both groups (40% for ischemic vs 41% of nonischemic, *P* = .92).

**Table 1:** Baseline characteristics

	Ischemic cardiomyopathy (n = 70)	Nonischemic cardiomyopathy (n = 172)	*P*‐value
Follow‐up, months, mean (SD)	10.2 (4.5)	9.9 (3.8)	.61
Age, mean (SD)	69 (9.0)	64 (12)	.002
Female	12 (17)	62 (36)	.004
BMI, mean (SD)	32(7.2)	33(8.1)	.31
Type of AF			
Paroxysmal AF	31 (47)	52 (34)	.18
Persistent AF	12 (18)	36 (23)	
Long‐standing Persistent AF	23 (35)	66 (43)	
CHA_2_DS_2_‐VASc (SD)	4.78 (1.24)	3.63 (1.61)	<.0001
Hypertension	67 (96)	134 (78)	.0008
Sleep apnea			
None	43 (61)	98 (57)	.32
Untreated	11 (16)	19 (11)	
Treated with noninvasive ventilation	16 (23)	54 (32)	
Diabetes	25 (36)	38 (22)	.03
COPD	10 (14)	17 (10)	.31
Prior stroke	10 (14)	25 (15)	.95
Anemia	5 (7.3)	14 (8.2)	.81
Valve disease			
Mitral regurgitation	12 (17)	21 (12)	.31
Mitral stenosis	0	2 (1.2)	1.0^a^
History of mitral valve replacement	0	19 (11)	.002^a^
Aortic stenosis	0	3 (1.7)	.56^a^
Aortic regurgitation	0	4 (2.3)	.33^a^
History of aortic valve replacement	5 (7.1)	13 (7.6)	.91
Left atrial diameter, cm, mean (SD)	4.6 (0.77)	4.6 (0.77)	.99
EF, %, mean (SD)	42 (12)	47(11)	.0007
EF ≥ 50%	27 (39)	110 (64)	.0004
Serum creatinine, mg/dL, mean (SD)	1.15 (0.30)	1.10 (0.55)	.48
Cardiovascular medications			
Beta‐blocker	58 (83)	132 (77)	.29
Calcium channel blocker	26 (37)	71 (41)	.55
Digoxin	8 (11)	26 (15)	.45
ACE inhibitor	31 (44)	77 (45)	.95
ARB	13 (19)	33 (19)	.91
Aldosterone antagonist	9 (13)	24 (14)	.82
Antiarrhythmic medications			
None	28 (40)	59 (34)	.28
Class IC	2 (2.9)	14 (8.1)	
Class III	40 (57)	99 (58)	
Anticoagulation at baseline			
Warfarin	55 (80)	137 (80)	.21
Dabigatran	6 (8.7)	25 (15)	
Rivaroxaban	5 (7.3)	8( 4.7)	

Note

Baseline characteristics, comorbidities, and medical therapies, stratified by heart failure etiology. Values are presented as n (%) unless otherwise specified.

Abbreviations: AF, atrial fibrillation; COPD, chronic obstructive pulmonary disease; EF, ejection fraction; SD, standard deviation.

a Fisher's exact test was applied as a result of small expected values.

### 3.2 Symptoms and quality of life

**Figure 1:**
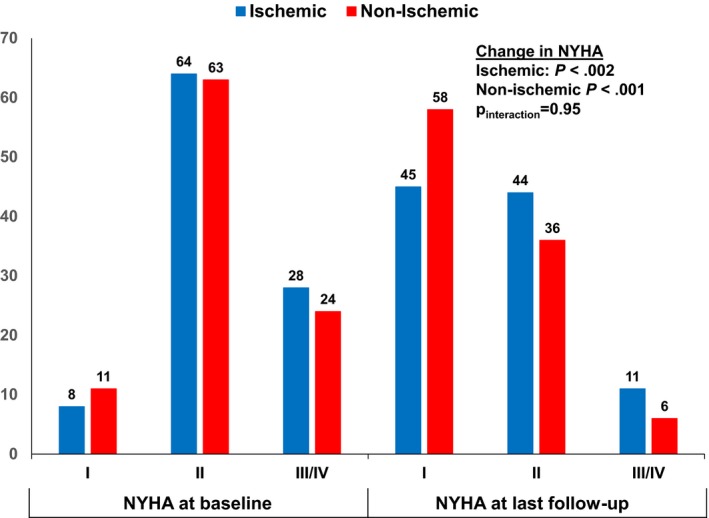
Change in NYHA class distribution from baseline to last follow‐up after ablation, stratified by heart failure etiology. NYHA = New York Heart Association functional classification with class I = no limitation in normal activity, class II = mild symptoms only in normal activity, class III = marked symptoms during daily activities but asymptomatic at rest, and class IV = severe limitations with symptoms at rest

**Figure 2:**
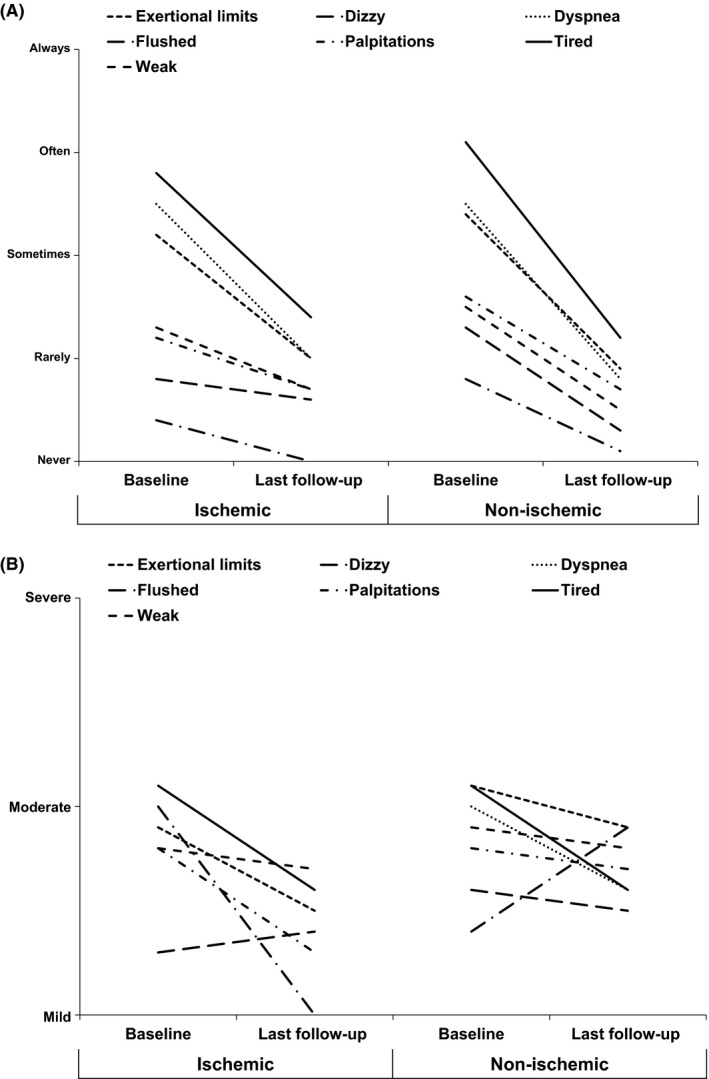
Change in mean MAFSI symptom frequency (A) and severity (B) from baseline to last follow‐up after ablation, stratified by heart failure etiology. MAFSI: Mayo AF Symptom Inventory. MAFSI symptom severity ranges from 0 = mild, 2 = moderate, to 4 = severe

Symptom status at baseline and at follow‐up are shown by NYHA class (Figure 1) and modified MAFSI scores (Figure 2). Nine patients were lost to follow‐up. Following catheter ablation there were significant improvements in NYHA classification in both ischemic and nonischemic patients (*P* < .002 for change in scores for each). The proportion of patients with NYHA class III/IV symptoms decreased from 28% to 11% in the ischemic group (60% reduction) and 24% to 6% (75% reduction) in the nonischemic group. However, changes in NYHA class were not different between ischemic and nonischemic patients (*P*_interaction_ = .95). Modified MAFSI scores demonstrated significant improvement in symptom frequency (Figure 2A) while there were mixed trends for symptom severity (Figure 2B).

### 3.3 Maintenance of sinus rhythm

Unadjusted clinical outcomes are shown in Table 2. Overall, freedom from recurrent atrial arrhythmia was 76.8% at 12‐months (n = 179/233). Freedom from recurrent atrial arrhythmia was 74.2% (n = 49/66) in those with ischemic cardiomyopathy vs 77.8% (n = 130/167) in those with nonischemic cardiomyopathy (*P* = .12). Freedom from antiarrhythmic drug use was 48% (n = 83/172) in those with ischemic HF vs 53% (n = 37/70) in those with nonischemic HF (*P* = .4). In unadjusted analyses, ischemic HF etiology was not significantly associated with either electrocardiographic (risk ratio [RR] 0.60, 95% confidence interval [CI] (0.32‐1.14) or symptomatic (RR 0.71, 95% CI 0.39‐1.31) recurrence at 12 months. In addition to routine clinic follow‐up and electrocardiograms, ambulatory monitoring with implantable device interrogation, 24‐hour Holter, event monitor, or implantable loop recorder) was performed in 167 patients overall (72.6%). There were no significant differences in the frequency of ambulatory monitoring in patients with ischemic (n = 54, 80%) and nonischemic (n = 113, 66%) cardiomyopathy (*P* = .36).

**Table 2:** Unadjusted outcomes by cardiomyopathy type

	Ischemic cardiomyopathy (n = 70)	Nonischemic cardiomyopathy (n = 172)	*P*‐value
Safety outcomes			
Periprocedural in‐hospital			
Access‐site bleeding	1 (1.4)	7 (4.1)	0.44
Stroke/TIA	1 (1.4)	1 (0.58)	0.50
Pericardial tamponade	0	0	—
Acute heart failure	3 (4.3)	8 (4.7)	1.0
Death	0	0	—
Effectiveness outcomes			
12‐Month Outcomes	N = 66	N = 167	
Recurrent atrial arrhythmia^a^	17 (26)	61 (37)	0.12
Antiarrhythmic use at 12 months			
None	37 (53)	83 (48)	0.38
Class Ic	1 (1.4)	9 (5.2)	
Class III	32 (46)	80 (47)	

Note

Values presented as n (%).

Abbreviations: AT/AF/AFL, atrial tachycardia/atrial fibrillation/atrial flutter; TIA, transient ischemic attack.

a Defined as AT/AF/AFL on 12‐lead electrocardiogram, ≥30 seconds on Holter or implantable device, or requiring cardioversion.

**Figure 3:**
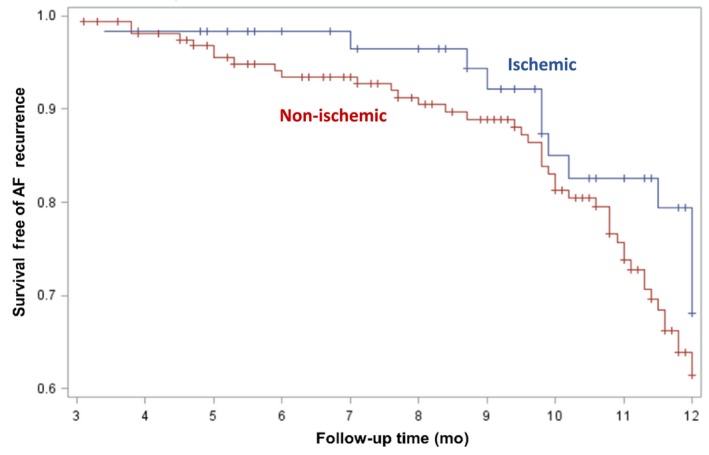
Kaplan‐Meier curve of arrhythmia‐free survival to 12 months according to heart failure etiology, with a 3‐month blanking period. AF recurrence was defined by electrocardiographic recurrence. Error bars represent 95% confidence limits

After adjustment, HF etiology was not significantly associated with freedom from recurrent atrial arrhythmia (Figure 3, ischemic vs nonischemic adjusted RR 0.63, 95% confidence interval [CI] (0.33‐1.19) or symptom recurrence at 12 months (ischemic vs nonischemic adjusted RR 0.71, 95% CI 0.37‐1.36). There were two patients in the ischemic cardiomyopathy group and 13 in the nonischemic cardiomyopathy group who underwent repeat ablation within 1 year.

### 3.4 Safety outcomes

In‐hospital, periprocedural adverse events were uncommon overall, and low numbers precluded meaningful comparison between heart failure etiology groups. Specifically, acute decompensated HF only occurred in 11 patients, including 4.3% (n = 3/70) in those with ischemic cardiomyopathy vs 4.7% (n = 8/172) in those with nonischemic cardiomyopathy (*P* = 1.0).

## 4 DISCUSSION

Results from randomized clinical trials demonstrate a robust benefit of AF catheter ablation in patients with systolic HF, including improvement in quality of life and reductions in HF hospitalization and mortality.^16^, ^21^, ^23^ However, little is known about which factors predict net clinical benefit and risk of arrhythmia recurrence following AF ablation in patients with concomitant HF. It has been hypothesized that patients with nonischemic cardiomyopathy may experience worse outcomes following AF ablation compared with patients with ischemic cardiomyopathy, because of a propensity for more extensive atrial myopathy. In this analysis, we report the outcomes of 242 consecutive patients with AF and concomitant HF undergoing catheter ablation at our institution. Our data confirm prior reports which demonstrate that patients with concomitant HF experience symptomatic improvement and favorable outcomes after AF ablation. Importantly we saw no evidence of attenuation of effectiveness or differential benefit between patients with nonischemic vs ischemic etiology of HF.

Despite the benefits of AF ablation in HF, there are unique challenges with ablation in the HF population. Patients with HF are more likely to have advanced forms of AF, develop congestion with periprocedural fluid administration, and have higher risks of periprocedural stroke compared to patients without HF. Our data confirm prior reports^11^, ^12^, ^13^, and further demonstrate that AF ablation can be performed safely in HF patients with favorable effectiveness. In our cohort, 77% were free from recurrent atrial arrhythmia at 12‐months with or without antiarrhythmic drug therapy.

Prior work has shown that in patients with AF and HF, EF improves by 11% after ablation (95% CI 7.1‐15.2).^24^ While higher recurrence rates are observed after ablation in patients with HF,^25^ patients with HF who achieve sinus rhythm may experience improved cardiovascular outcomes. The AATAC clinical trial compared amiodarone vs catheter ablation in patients with moderate to severe HF (NYHA class II‐III) and an EF ≤ 40%.^23^ Patients randomized to ablation had greater maintenance of sinus rhythm (70% vs 34%), lower hospitalization (31% vs 57%), and numerically lower mortality (8% vs 18%). The CASTLE‐AF investigators randomized patients with systolic HF (LVEF ≤ 35), NYHA class II‐IV symptoms, and paroxysmal or persistent AF on optimal medical therapy to medical therapy or AF ablation. Following a mean follow‐up duration of 37.6 ± 20.4 months, patients receiving catheter ablation were less likely to experience the primary end point of death or HF hospitalization (HR 0.062, 95% CI 0.43‐0.87, *P* = .007).^16^ Fewer patients receiving catheter ablation died during follow‐up than medical therapy (HR 0.49, 95% CI 0.29‐0.84, *P* = .009).^16^ There was also a statistically nonsignificant trend toward improved clinical outcomes in patients in the CABANA trial with HF.^26^ The result of CABANA, bolstered by other smaller randomized trials,^27^ transformed the role of catheter ablation in AF in HFrEF from hypothetical to guideline‐based, as reflected by a class IIB, level of evidence B recommendation in the 2019 Focused Update of the AHA/ACC/HRS Guidelines for Management of Patients with Atrial Fibrillation.^28^

Patients with HF have more advanced atrial myopathy and HF is a well‐documented risk for AF progression.^29^ The etiology of HF has been demonstrated to influence response rates and outcomes following other electrophysiologic interventions in HF, including catheter ablation of ventricular tachycardia^17^ and cardiac resynchronization therapy.^18^ Patients with nonischemic cardiomyopathy may be more likely to have more advanced atrial myopathy and thus experience higher recurrence rates and more symptoms after AF ablation compared with patients that have ischemic cardiomyopathy. The DECAAF (Association of Atrial Tissue Fibrosis Identified by Delayed Enhancement MRI and Atrial Fibrillation Ablation) study demonstrated that atrial fibrosis assessed using delayed gadolinium enhanced MRI is predictive of AF recurrence following ablation.^30^ However, the 329 patient DECAAF study included a relatively few patients with congestive heart failure (n = 15) and coronary artery disease (n = 26) and found no statistical association between either factor and atrial fibrosis.^30^

Importantly, we did not observe differences in outcomes between patients with ischemic cardiomyopathy vs those with nonischemic cardiomyopathy. A systematic review has shown greater improvement in ventricular function in cohorts with less ischemic disease undergoing catheter ablation for AF.^24^ This finding was consistent in a recent, smaller cohort (n = 100) in which improved AF control was observed in patients with idiopathic cardiomyopathy undergoing ablation, compared to patients with known causes of structural heart disease.^31^ Similar to our study findings, there was no difference in outcomes between patients with ischemic and nonischemic cardiomyopathy in CASTLE‐AF.^27^ It is unclear whether differential rates of recurrence and response among distinct subgroups of NICM could be related to the prevalence of non‐PVI triggers or atrial substrate. Several forms of nonischemic cardiomyopathy, particularly hypertrophic cardiomyopathy and cardiac amyloid, are known to predispose to more recalcitrant AF despite ablation.^32^ Left atrial electroanatomic maps of patients with cardiac amyloid demonstrate extensive atrial low voltage, and AF ablation in these patients is associated with a particularly high rates of recurrence.^33^

Outside of recurrence rates, there are few published data on symptom status in patients undergoing AF ablation with ischemic vs nonischemic cardiomyopathy. While LVEF is an important surrogate endpoint, quality of life is much more closely tied to functional status. Our data demonstrate a clear improvement in functional status following ablation, as indicated by both by HF and AF assessments. The improvements in symptom frequency and functional status were not different in patients with ischemic and nonischemic HF. There are several possible explanations for the similar effectiveness observed in both ischemic and nonischemic HF patients. First, it is possible that AF ablation is equally durable in patients with ischemic and nonischemic cardiomyopathy despite differences in the underlying substrate or trigger density. It is also possible that short‐term and immediate‐term follow‐up is similar, but with longer follow‐up and more opportunity for substrate progression, differences would have emerged. However, based upon our data, the prognosis following AF ablation appears to be similar in those with ischemic vs nonischemic cardiomyopathy. Both patients with ischemic and nonischemic cardiomyopathy appear to do well after AF ablation with similar risk of recurrence at one year.

Further investigation into which factors (including the specific etiology of nonspecific cardiomyopathy) predict clinical improvement following AF ablation in HF is needed. In patients with nonischemic cardiomyopathy, the presence and extent of ventricular scar as assessed by ventricular late gadolinium enhancement (LGE) may be important. The CAMERA‐MRI study assessed LGE on preablation MRI in 301 patients with idiopathic cardiomyopathy undergoing AF ablation.^34^ Patients with no LGE on MRI had greater LVEF improvement (10.7%, *P* = .0069) at 6 month follow‐up.^34^ Patients with less severe left ventricular dysfunction (LVEF 25%‐35%) received more benefit from AF ablation than those with severe LV dysfunction (LVEF < 25%) in CASTLE‐AF.^16^ Detailed analysis of the HF population in the CABANA trial have not yet been published, but will hopefully provide additional insights.

### 4.1 Limitations

This is a single‐center, retrospective, observational study, which may limit generalizability. Furthermore, the diagnosis of heart failure with preserved ejection fraction was made clinically, and formal diagnostic criteria for this entity vary even in the context of carefully conducted clinical trials. However, our results in the overall HF cohort parallel those seen in other published reports. Another limitation is that the procedures represent a relatively homogenous approach to pulmonary vein isolation, and did not include cryoablation. Follow‐up for recurrence with ambulatory monitoring (eg Holter monitor) was done based on clinical and symptom status rather than routine, as these procedures were performed for the primary indication of symptom relief. Asymptomatic patients did not undergo a routine monitoring outside of routine ECG follow‐up unless they had an implanted device. Furthermore, the study period predates important advances in AF ablation, including contact force‐sensing catheters, rotor mapping, and high‐density electroanatomic mapping systems. However, it is not likely that these technical improvements would expect to impact one etiology of HF more than the other. Finally, despite the use of extensive multivariable adjustment, we cannot exclude the presence of unmeasured or residual confounding that might have impacted the results.

## 5 CONCLUSIONS

Consistent with prior experience, catheter ablation for AF in patients with concomitant HF is associated with substantial improvement in symptoms, functional status, and freedom from recurrent atrial arrhythmia. While the underlying myopathic processes may be different in patients with ischemic and nonischemic HF, we found no significant differences in outcomes according to HF etiology following catheter ablation of AF. Unlike other electrophysiologic interventions, patients with ischemic and nonischemic cardiomyopathy appear to derive a similar magnitude of benefit following AF ablation. Further investigations are needed to determine which subgroups of patients with concomitant AF and HF are most likely to benefit from catheter ablation.

## DISCLOSURES

E. Black‐Maier: None. BA Steinberg: Fellowship Support; Medtronic, Inc, St. Jude Medical, Biotronik, Boston Scientific Corp. Fellowship Support; Biosense Webster, Inc.; Boston Scientific Corp., Janssen Pharmaceuticals. Other; Bristol Meyers Squibb. K. Trulock: None. F. Wang: None.. Y. Lokhnygina: None. W. O 'Neal: None. SM Al‐Khatib: None. BD Atwater: Research Grants; St. Jude Medical. JP Daubert: consulting fees/honoraria from Medtronic, St. Jude Medical, Boston Scientific, Sorin Group, and CardioFocus' research grants from Boston Scientific, Biosense Webster, Medtronic, and Gilead Sciences. C. Frazier‐M ills: None. DD Hegland: None. KP Jackson: None. LR Jackson: None. JI Koontz: None. RK Lewis: None. AY Sun: None. KL Thomas: None. TD Bahnson: Research Grants: Medtronic; St. Jude Medical; Consultant to Boehringer Ingelheim, ChanRX, Sequel Pharma, and Sanofi‐Aventis. JP Piccini: research grants from Abbott, American Heart Association, Bayer, Boston Scientific, Gilead, Janssen Pharmaceuticals, NHLBI, and Philips and consultant fees from Abbott, Allergan, ARCA Biopharma, Biotronik, Boston Scientific, Johnson & Johnson, LivaNova, Medtronic, Milestone, Sanofi, and Philips.
