# Supplementary material for: Effectiveness of catheter ablation of atrial fibrillation according to heart failure etiology
Source: J Arrhythm. 2020 Jan 8;36(1):84–92. doi: 10.1002/joa3.12291 (PMC7011840; doi:10.1002/joa3.12291)
Supplement: Supplementary file 1 [file JOA3-36-84-s001.docx]

Table S1. Ablation characteristics by cardiomyopathy type.

|  | Ischemic Cardiomyopathy (n=70) | Non-Ischemic Cardiomyopathy (n=172) | P-value |
| --- | --- | --- | --- |
| Total procedure duration, minutes, mean (SD) | 259 (82) | 243 (74) | 0.13 |
| RF Total duration, minutes, mean (SD) | 58 (24) | 56 (27) | 0.60 |
| Total fluoroscopy duration, minutes, mean (SD) | 51 (27) | 54 (27) | 0.44 |
| Additional Lesion Sets |  |  |  |
| Mitral Isthmus Line | 8 (11) | 20 (12) | 0.96 |
| LA Roof Line | 28 (40) | 70 (41) | 0.92 |
| Substrate (i.e., CAFÉ) | 12 (17) | 43 (25) | 0.19 |
| Coronary sinus ablation | 8 (11) | 26 (15) | 0.45 |
| Non-LA Ablation (e.g., SVC) | 14 (20) | 29 (17) | 0.56 |
| Concomitant CTI Ablation | 10 (14) | 23 (13) | 0.85 |
| Antiarrhythmic at Discharge |  |  | 0.13 |
| None | 9 (13) | 25 (15) |  |
| Class Ic | 1 (1.4) | 14 (8.1) |  |
| Class III | 60 (86) | 133 (77) |  |

SD: standard deviation; CAFÉ: complex atrial fractionated electrograms; SVC: superior vena cava; CTI: cavotricuspid isthmus

Values are presented as n (%) unless otherwise specified.
